# Prolonged Copper Supplementation Modified Minerals in the Kidney, Liver and Blood, and Potentiated Oxidative Stress and Vasodilation of Isolated Aortic Rings in Young Wistar Rats

**DOI:** 10.3390/nu16193230

**Published:** 2024-09-24

**Authors:** Klaudia Kitala-Tańska, Anetta Hanć, Jerzy Juśkiewicz, Michał Majewski

**Affiliations:** 1 Department of Pharmacology and Toxicology, Faculty of Medicine, University of Warmia and Mazury in Olsztyn, 10-082 Olsztyn, Poland; klaudia.kitala@wp.pl; 2 Department of Trace Analysis, Faculty of Chemistry, Adam Mickiewicz University, 61-614 Poznań, Poland; anetta.hanc@amu.edu.pl; 3 Division of Food Science, Institute of Animal Reproduction and Food Research, Polish Academy of Sciences, 10-748 Olsztyn, Poland; j.juskiewicz@pan.olsztyn.pl

**Keywords:** 1400 W, aortic rings, copper, COX, iNOS, NS-398, SC-560, trace elements, vascular studies

## Abstract

Background: Previous studies have highlighted that copper supplementation at 200% of the recommended daily dietary allowance modified vascular contraction and relaxation through increased reactive oxygen species (ROS) and prostaglandin formation, which modified the antioxidant status of middle-aged Wistar rats. Methods: In this study, young (1 month old) male Wistar rats (*n*/group = 10) received a diet supplemented with 6.45 mg copper/kg (100% of daily recommendation—Group A) for 8 weeks. The experimental group received 12.9 mg copper/kg of diet (200% of the daily recommendation—Group B). Results: Experimental supplementation with 200% copper modified the copper concentration in the blood (1.21-fold, *p* = 0.04), liver (1.15-fold, *p* = 0.032), and kidneys (1.23-fold, *p* = 0.045), potentiated the ROS formation in the aortic rings, and enhanced the sensitivity of the aortic rings to the vasodilator acetylcholine. We observed an increased participation of nitric oxide (NO) derived from inducible NO synthase (iNOS) in vascular contraction and a decreased net effect of vasodilator prostanoids derived from cyclooxygenase-2 in vascular relaxation. In rat kidneys, the concentrations of potassium (1.08-fold, *p* = 0.001) and iron (1.13-fold, *p* = 0.046) were higher, while, calcium (0.88-fold, *p* = 0.001) and chromium (0.77-fold, *p* = 0.005) concentrations were lower. In the rat liver, magnesium (1.06-fold, *p* = 0.012) was higher. No differences were observed in the concentrations of sodium, zinc, manganese, selenium, cobalt, molybdenum, and vanadium. The antioxidant activity of water- and lipid-soluble compounds; total antioxidant status in the blood; and superoxide dismutase, catalase, and malondialdehyde levels in the heart did not change. Conclusions: In young rats, prolonged supplementation with 200% copper had a lesser effect than anticipated on oxidative stress and vascular reactivity. Detailed data on the status of trace elements and their interactions in patients of different age groups are strongly required for effective nutritional and therapeutic intervention.

## 1. Introduction

Understanding the impact of copper supplementation in different age groups and the impact of aging on copper pharmacokinetics remains a topic of ongoing research due to inaccurate findings from previous studies.

Copper is a vital dietary element for mammals, and therefore, insufficient or excessive consumption of this trace element can have negative effects on well-being. With a high copper intake, the body’s ability to absorb copper decreases, while the elimination of copper increases. In contrast, when copper intake is low, the body reduces the excretion of copper through the bile and retains more copper [1]. Furthermore, considering the natural tendency of copper to enhance the generation of detrimental oxygen free radicals, excessive amounts of copper have the potential to cause tissue damage and subsequent pathological consequences [2]. The inequitable production and elimination of reactive oxygen species (ROS) may result in the buildup of intermediate ROS products, which are considered harmful and have the potential to trigger oxidative stress [3]. Copper also has crucial functions in a multitude of physiological processes. These processes include respiration, removal of harmful free radicals, regulation of iron and oxygen metabolism, formation of connective tissues, maturation of the extracellular matrix, production of energy, synthesis of neuropeptides, and facilitation of neuroendocrine signaling [4]. The potential for oxidation exhibited by copper may contribute to its toxicity in instances of excessive ingestion. At elevated concentrations, copper has been observed to induce oxidative harm to biological systems through the peroxidation of lipids and other macromolecules [5].

Changes in copper pharmacokinetics may occur with aging. Available data point to no variations in copper absorption across different age groups; however, it was observed that serum copper levels were notably elevated in elderly men [6]. In addition, age had a significant impact on the measurement of ceruloplasmin [7].

Our aim was to analyze the impact of prolonged copper supplementation with 200% of the recommended daily dietary allowance on mineral concentration, oxidative stress, and cardiovascular function in young rats.

## 2. Materials and Methods

### 2.1. Substances

The following chemicals were obtained from Sigma–Aldrich (St. Louis, MO, USA), acetylcholine chloride, noradrenaline hydrochloride, selective inducible nitric oxide synthase (iNOS) inhibitor (1400 W), selective cyclooxygenase-2 (COX-2) inhibitor (NS-398), and selective COX-1 inhibitor (SC-560). NS-398 and SC-560 were dissolved in DMSO, and 1400 W was dissolved in methanol. Noradrenaline was dissolved in a solution of NaCl and ascorbic acid at concentrations of 0.9% and 0.01% *w*/*v*, respectively. The concentration of the solvent was less than 0.01% (*v*/*v*). The solutions were stored at −20 °C. On the day of the experiment, the solutions were diluted in Krebs-Henseleit solution (KHs in mM: NaCl—115; CaCl_2_—2.5; KCl—4.6; KH_2_PO_4_—1.2; MgSO_4_—1.2; NaHCO_3_—25; glucose—11.1) [8].

### 2.2. Animals and Diet

A group of 20 male Wistar Han rats, aged 4 weeks, were provided with a standard rat diet (Diet A) that included 6.45 mg/kg copper, as per the guidelines outlined in references [8,9]. For an additional duration of 8 weeks, the rats in Group B (*n*/group = 10) were fed a diet containing 12.9 mg Cu/kg (200% of the recommended daily dietary quantity of copper), while those in Group A were fed a standard rat diet (Diet A). A copper carbonate sample, which had a purity of at least 99%, was acquired from Poch (Gliwice, Poland). The rats were housed in accordance with a previously described housing method [8]. Unrestricted access to food and tap water (a copper concentration of 0.35 mg/L) was provided. Blood samples were obtained from the caudal vena cava of anesthetized animals. Anesthesia was administered through intraperitoneal injections of ketamine and xylazine at dosages of 100 mg/kg and 10 mg/kg of body weight, respectively.

### 2.3. Body Weight and Body Composition

A Bruker Minispec LF (Ettlingen, Germany) was used to accurately measure fat, fluid, and lean tissues in rats using time domain−nuclear magnetic resonance (TD-NMR) [10].

### 2.4. Vascular Reactivity Studies

As previously described, the aortic rings were incubated in 5 mL chambers (Graz, Barcelona, Spain) under a preload tension of 1 g (FT20, TAM-A, Hugo Sachs Elektronik, March, Germany) [11]. The functional integrity of the aortic rings was assessed using high concentrations of the vasoconstrictor KCl (75 mM) and the vasodilator acetylcholine (10 µM). In addition, the aortic rings were subjected to a 30 min preincubation with various substances, including a selective COX-1 inhibitor (SC-560, 10 µM), a selective COX-2 inhibitor (NS-398, 10 µM), and an iNOS inhibitor (1400 W, 1 µM). The vasodilatory response was analyzed by adding cumulative concentrations (CCs) of acetylcholine (ranging from 0.1 nM to 10 µM) to the incubation chambers [8]. The response to noradrenaline (0.1 µM) was also studied.

### 2.5. The Langendorff Heart Studies

The isolated heart studies were conducted using the Langendorff system equipped with ISOHEART software 73-0161 (Hugo Sachs Elektronik, March, Germany), following the methodology outlined in a previous study [8]. Balloon inflation was implemented to establish a diastolic pressure of 8–10 mmHg. The measurements included the heart rate, mean arterial pressure, diastolic pressure, and systolic pressure.

### 2.6. Analysis of Minerals in Rat Liver and Kidneys

#### 2.6.1. Sample Preparation

Each analyzed liver and kidney sample was cut into small pieces using a ceramic knife and mixed to homogenize the sample. Three independent samples were prepared for each organ (*n* = 3). Approximately 0.2 g of the wet samples (kidney ~32% and liver ~40% water), 3 mL of 65% nitric acid, and 0.5 mL of 30% hydrogen peroxide were used for digestion. The digestion proceeded in two steps: permineralization for 3 h and mineralization in a closed system. The samples were mineralized using a microwave mineralizer (EthoS One, Milestone, Sorisole, Italy). The heating program during mineralization in the closed system was as follows: 20 min ramp time to 220 °C, 30 min at 220 °C, and 20 min of cooling. The power of the entire process was 1500 W. The digested samples were diluted with distilled water (Direct-Q-3 UV, Merck, Darmstadt, Germany) to a volume of 50 mL. The certified reference material ERM, BB184 Bovine Muscle (IRMM, Geel, Belgium), was used to evaluate the accuracy of the measurements and was prepared in the same way as the analyzed samples.

#### 2.6.2. Elements Measurements

Inductively coupled plasma–mass spectrometry (ICP-MS 7100x Agilent, Santa Clara, CA, USA) was used for analysis. The instrumental parameters were automatically optimized using Tuning Solution (Agilent). For the reduction of interferences, helium mode was used. The interferences were reduced by using helium mode and an internal standard solution containing 10 µg/L Rh and Tb. Calibration solutions were prepared by the appropriate dilution of a 10 mg/L multielement standard solution (ICP Standard, Perkin Elmer, Darmstadt, Germany). Calibration curves were constructed in the range of 0.01–100 µg/L. High purity argon (99.999%) was used as the nebulizer, auxiliary, and plasma gas for the ICP-MS (Messer, Chorzów, Poland).

#### 2.6.3. Quality Assessment

The validity of the analytical method was assessed by analyzing the certified reference material ERM BB184 Bovine Muscle (IRMM, Geel, Belgium). Validation parameters, such as linearity, precision, limit of detection (LOD), and trueness were evaluated. The linearity of the calibration curves, calculated as the correlation coefficient R, was greater than 0.9996 for all the analytes. The LOD was defined as 3.3 s/b, where s is the standard deviation corresponding to 10 blank injections and b is the slope of the calibration graph. The LODs were as follows: 0.008 µg/g for Cu, 0.012 µg/g for Zn, 0.075 µg/g for Se and 0.214 µg/g for Fe. The precision values were calculated as the coefficient of variation (CV) (%) and ranged from 1.2% to 3.4% for all the elements. Trueness was evaluated by applying the certified reference material and was expressed as recovery values (%) ranging from 95% to 103%.

### 2.7. Analysis of Minerals in Rat Blood

The concentration of elements in rat serum was determined by Atomic Absorption Spectroscopy, as previously described [9].

### 2.8. Oxidative Stress and Antioxidant Parameters

The blood serum total antioxidant status (TAS) was measured using a Randox kit through a spectrophotometric method. The antioxidant capacity of water (ACW) and lipid (ACL) soluble compounds was determined using the photo-chemiluminescence method, which detects generated free radicals that are removed with the use of the antioxidants ascorbic acid and Trolox. Malondialdehyde (MDA) was quantified with a fluorometric assay kit (ab118970), according to the manufacturer’s instructions; catalase (CAT) and superoxide dismutase (SOD) activities were measured using diagnostic kits from Oxis International, Inc. (Portland, OR, USA) and Ransel and Ransod colorimetric diagnostic kits from Randox (Warsaw, Poland) [8].

Blood serum was analyzed using commercial ELISA kits to determine the levels of COX-1, COX-2, heme oxygenase-1 (HO-1), endothelial nitric oxide synthase 3 (NOS3), glyceraldehyde 3-phosphate dehydrogenase (GAPDH), and intercellular adhesion molecule 1 (ICAM-1), see Table S1 [8]. A Thermo Scientific microplate reader (Varioskan LUX, Bremen, Germany) was used to measure the absorbance of the ELISA test plate at a wavelength of κ = 450 nm.

The levels of nitric oxide (NO), superoxide anion (O_2_^•−^), and hydrogen peroxide (H_2_O_2_) in situ were assessed using diaminofluorescein (DAF), dihydroethidium (DHE), and 2′,7′-dichlorofluorescein diacetate (DCF), following the methodology described in a previous study [9]. The cells were incubated with the nuclear dye 4′,6-diamidino-2-phenylindole (DAPI, 10 µg/mL) in the following combinations: DAF + DAPI, DHE + DAPI, and DCF + DAPI. The incubation was carried out for 30 min in an oven at 37 °C. Fluorescence images were acquired using a Leica (TCS ST2DM IRE2) laser scanning confocal microscope at wavelengths of 568 nm and 410–475 nm for the DAPI dye.

### 2.9. Data Analysis and Statistics

Vascular contraction in response to noradrenaline (0.1 μM) was quantified in milligrams of tension, while vascular relaxation in response to acetylcholine was measured as a percentage of the contractile response to noradrenaline. A log agonist vs. response (nonlinear regression model) was employed to analyze the cumulative concentration–response curves (CCRCs). Comparisons between groups were conducted using either a parametric test (*t*-test) or a nonparametric test (Mann-Whitney U test or Kruskal-Wallis test), with a sample size of *n* = 10 rats/group. The CCRCs were subjected to analysis via two-way ANOVA with Šídák’s multiple comparisons test. The results are presented as the means ± SEMs (for CCRCs) or means ± SDs. The significance level was set at * *p* ≤ 0.05.

## 3. Results

### 3.1. Rat Characteristics

The control group (Group A) received 100% copper (6.45 mg copper/kg of diet) and was compared to the experimental group (Group B), which received 200% copper (12.9 mg copper/kg of diet).

The amount of daily food consumed did not differ between the groups. Body weight gain was not affected. The weights of the internal organs (the heart, liver, kidneys, spleen, and brain) did not change. Body fat and lean body parts were not modified.

### 3.2. Minerals

#### 3.2.1. Serum

The copper concentration was higher in the rat blood serum (mg/L): 1.01 ± 0.31 vs. 1.22 ± 0.24 (1.21-fold, *p* = 0.04). The calculated copper-to-zinc ratio was also higher 1.02 ± 0.23 vs. 1.42 ± 0.356 (1.39-fold, *p* = 0.04). 

No significant differences were observed in the zinc and selenium concentrations between the studied groups (Table S2).

#### 3.2.2. Liver

The concentrations of copper (1.15-fold, *p* = 0.032) and magnesium (1.06-fold, *p* = 0.012) were higher in the study group. The calculated copper-to-zinc ratios were also higher (1.09-fold, *p* = 0.014); see Table S2.

No significant differences were observed in potassium, iron, calcium, chromium, sodium, zinc, manganese, selenium, cobalt, molybdenum, and vanadium concentrations between the studied groups, see Table S2.

#### 3.2.3. Kidney

The concentrations of copper (1.23-fold, *p* = 0.045), iron (1.13-fold, *p* = 0.046), and potassium (1.08-fold, *p* = 0.001) were higher in the study group. The calculated copper-to-zinc ratio was also higher (1.16-fold, *p* = 0.05), while the concentrations of calcium (0.88-fold, *p* = 0.001) and chromium (0.77-fold, *p* = 0.005) were lower.

No significant difference exists in magnesium, sodium, zinc, manganese, selenium, cobalt, molybdenum, and vanadium concentrations between the studied groups, see Table S2.

### 3.3. Oxidative Stress and Antioxidant Parameters

The amounts of NO, O_2_^•−^, and H_2_O_2_ were higher in the rat aortic rings. NO: 4265 ± 874 vs. 6342 ± 987 (1.49-fold, *p* = 0.04); O_2_^•−^: 3254 ± 253 vs. 5324 ± 1034 (1.64-fold, *p* = 0.023); and H_2_O_2_: 5765 ± 753 vs. 7789 ± 1075 (1.35-fold, *p* = 0.015).

No significant differences were observed in the ELISA results for COX-1, COX-2, GAPDH, ICAM-1, HO-1, and eNOS in the rat serum between the studied groups (Table S2).

No significant differences were observed in TAS, ACW, ACL, MDA, SOD, and CAT levels between the studied groups in the rat serum or heart (Table S2).

### 3.4. The Isolated Perfused Heart

No significant differences were observed in the cardiac contractile strength and heart rate between the studied groups.

### 3.5. Vascular Contraction

**Figure 1:**
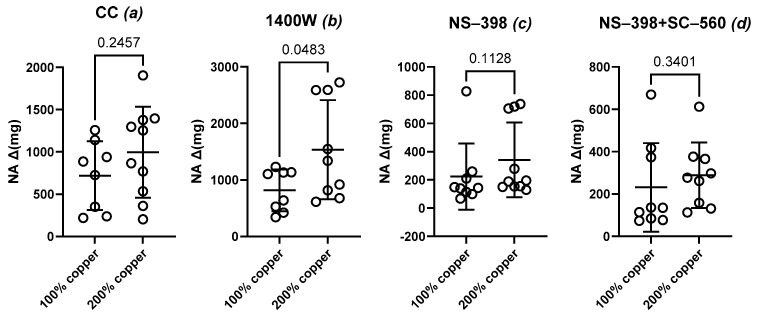
Vasoconstriction in response to noradrenaline (NA) either under the control conditions (**a**) or when subjected to preincubation with the inducible nitric oxide synthase (iNOS) inhibitor (1400 W) (**b**), the selective cyclooxygenase-2 (COX-2) inhibitor (NS-398) (**c**), and the sum of selective COX-2 and selective COX-1 (SC-560) inhibitors (**d**); *t*-test with *p* ≤ 0.05. Preincubation with the selective iNOS inhibitor modified vasoconstriction.

**Figure 2:**
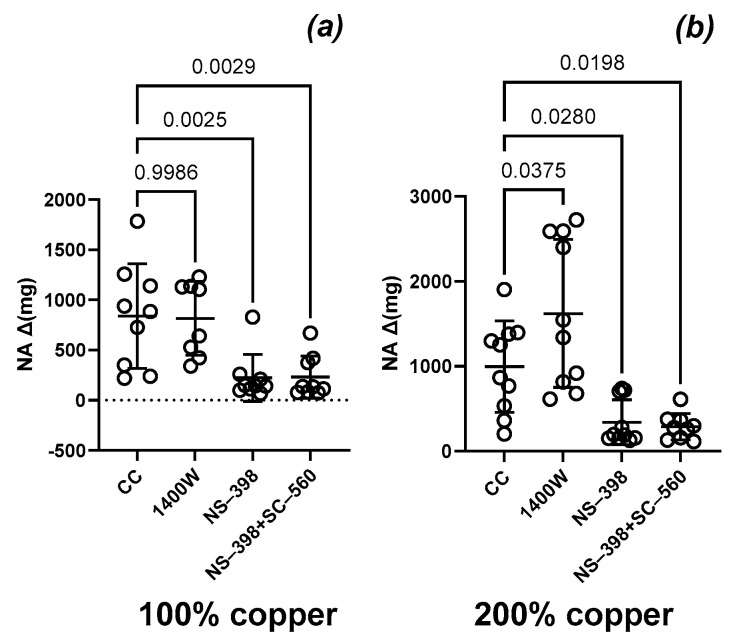
Vasoconstriction in response to noradrenaline (NA) in the control group with 100% copper (**a**) and the experimental group with 200% copper (**b**). Aortic rings were preincubated with the inducible nitric oxide synthase (iNOS) inhibitor (1400 W), the selective cyclooxygenase-2 (COX-2) inhibitor (NS-398), and the sum of selective COX-2 and selective COX-1 (SC-560) inhibitors. Two-way ANOVA with *p* ≤ 0.05. Preincubation with the selective iNOS inhibitor modified vasoconstriction.

We observed no significant difference in the NA-induced contraction between the studied groups under the control conditions (CC, Figure 1a) or when the aortic rings were preincubated with either selective COX-1 inhibitor (Figure 1c) or COX-1 plus COX-2 inhibitors simultaneously (Figure 1d). A selective COX-2 inhibitor (NS-398) decreased vascular contraction in both groups (Figure 2a,b), and a similar effect was observed when COX-1 and COX-2 were blocked simultaneously (Figure 2a,b).

Preincubation with the selective iNOS inhibitor potentiated vasoconstriction compared to that in the control group (Figure 1b). The effect of the selective iNOS inhibitor was also significantly greater in experimental group B, but not in control group A (Figure 2a,b).

### 3.6. Vascular Relaxation

**Figure 3:**
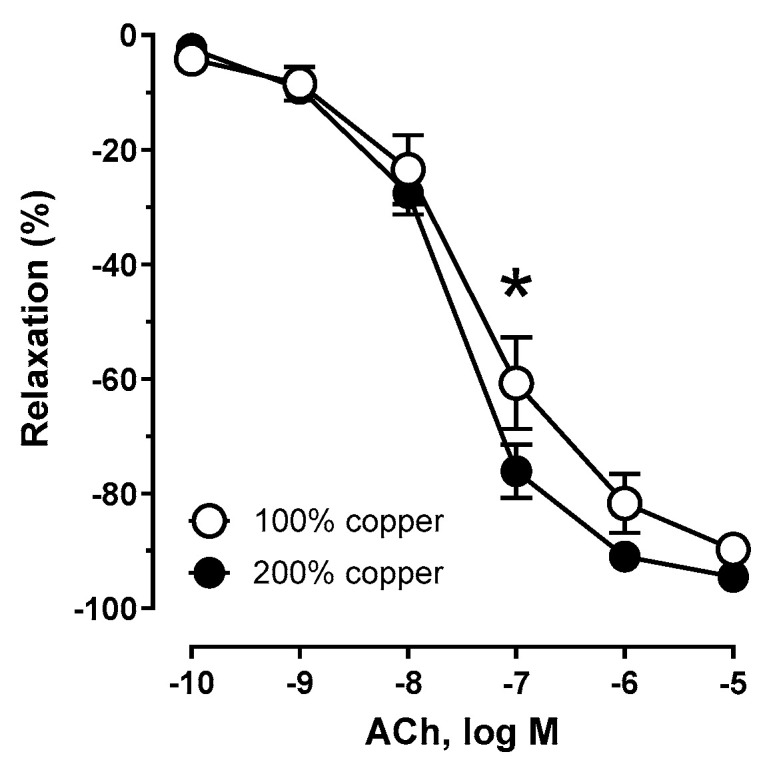
Vasodilator response to acetylcholine (ACh) of the isolated aortic rigs from copper-supplemented rats. Two-way ANOVA with * *p* ≤ 0.05. Supplementation with 200% copper potentiated the sensitivity to ACh.

**Figure 4:**
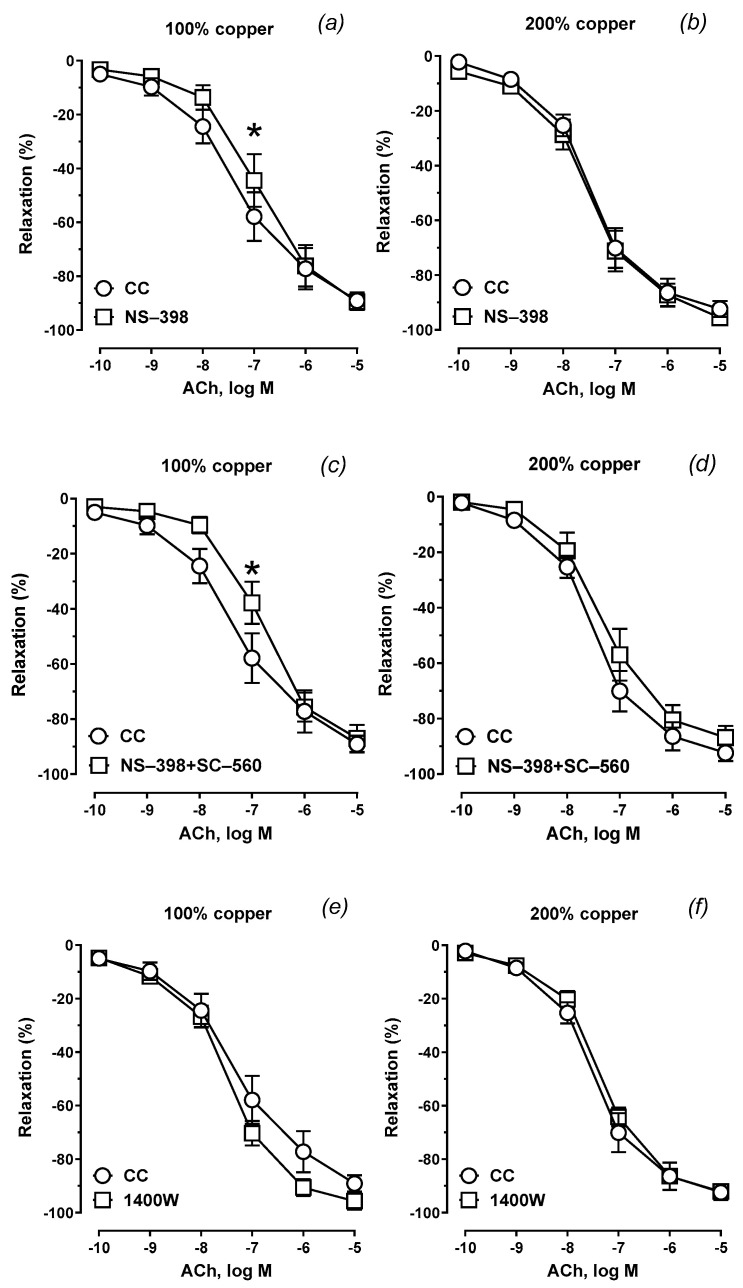
Vasodilator response to acetylcholine (ACh) of the isolated aortic rigs from copper-supplemented rats. Aortic rings were preincubated with the selective cyclooxygenase-2 (COX-2) inhibitor (NS-398) (**a**,**b**), selective COX-1 (SC-560) and COX-2 inhibitors (**c**,**d**), and inducible nitric oxide synthase (iNOS) inhibitor (1400 W) (**e**,**f**). Two-way ANOVA with * *p* ≤ 0.05. Preincubation with a selective COX-1 inhibitor and a COX inhibitor, but not with an iNOS inhibitor, attenuated the vasodilator response to acetylcholine.

Supplementation with 200% copper potentiated the sensitivity to ACh compared to 100% copper (the control group), as shown in Figure 3 and Table 1. Preincubation with a selective COX-1 inhibitor (Figure 4a,b) and COX inhibition (COX-1 + COX-2 inhibitors, Figure 4c,d) attenuated the vasodilator response to acetylcholine. This effect was not observed with the iNOS inhibitor (Figure 4e,f).

## 4. Discussion

**Figure 5:**
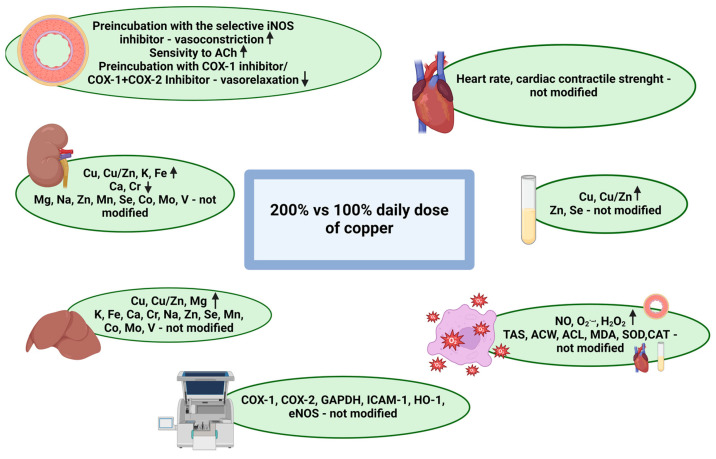
Summary of the results. Created in BioRender. Kitala-Tańska, K. (2024) www.BioRender.com/w45p634 [12].

Significant changes were observed in mineral concentrations in the kidney, liver, and blood; vascular reactivity; and oxidative stress markers. The results are summarized in the diagram (Figure 5).

Copper exerts a regulatory influence on the activity of nitric oxide synthase (NOS) and guanylyl cyclase (GC) in vascular structures [13]. The modified process of synthesizing and releasing vasoactive factors can potentially play a role in the impairment of blood vessel function, ultimately resulting in the emergence of cardiovascular disorders (CVDs), such as atherosclerosis, hypertension, and ischemia. To effectively develop prevention and treatment strategies, it is imperative to comprehend the mechanisms by which elevated serum copper levels impact the risk of atherosclerotic CVD. There are several ways in which high serum levels of copper can potentially increase the risk of atherosclerotic CVD. One possible mechanism is through the oxidative modification of low-density lipoprotein cholesterol and the formation of free radicals, which can contribute to the development of atherosclerosis [14]. Another pathway involves inflammation, as copper is closely associated with ceruloplasmin, an acute-phase reactant [14]. Copper overload-induced T cell apoptosis and proliferation defects [15]. Additionally, copper has been linked to insulin resistance and the pathogenesis of diabetes, which are major risk factors for coronary heart disease (CHD) [16]. Finally, high levels of copper can lead to luminal narrowing of the arteries as it promotes the expansion of the arterial neointima, which is primarily composed of copper-containing extracellular matrix molecules [17]. High levels of copper can lead to the aging of endothelial cells and the release of a senescence-associated secretory phenotype. It can also impair the function of various blood vessels, including the production of NO, cellular movement and growth, and ability to withstand toxins within cells [18]. 

We have shown that the vasoconstrictor response to noradrenaline was similar across the two studied groups and that neither selective COX-2 inhibition nor COX inhibition (simultaneous COX-1 and COX-2 inhibition) modified the contractile response. In contrast to our research (with 1.2 mg of copper/L of rat serum detected), other in vitro studies demonstrated that copper concentrations of 10 and 16 µM (0.635 and 1.016 mg/L, respectively) exhibited a dose-dependent inhibitory effect on phenylephrine-induced contractions in isolated rings of the rat thoracic aorta [19]. A subsequent in vitro study demonstrated that copper pretreatment blocked vasoconstriction induced by noradrenaline, both dependently and independently of the bioavailability of NO. This suggests that the effect of copper pretreatment is attributed to a mechanism other than NO [13]. Earlier studies discovered that increased copper intake in middle-aged rats led to reduced vasoconstriction when exposed to noradrenaline. Additionally, preincubation with a prostaglandin F2α analog (which is a selective FP receptor antagonist) rendered the vasoconstrictor response insignificant across groups, suggesting that FP receptors are involved in reduced contraction in copper-supplemented middle-aged rats [9]. In the presented study, this was not the case, and surprisingly, a selective iNOS inhibitor potentiated vascular contraction in response to noradrenaline, indicating that increased production of NO derived from iNOS constitutes a compensatory mechanism in copper-supplemented rats. Furthermore, Cuzzocrea et al. confirmed the presence of a complex relationship between copper homeostasis and NO metabolism. Similar to NO, copper can also have either beneficial or toxic effects. This is dependent on the specific site and modality of action, such as the isoform involved, enzyme activity, or expression [20].

The vasodilatory response to acetylcholine tended to potentiate in the experimental group. It is worth mentioning that the increase in vasodilation in middle-aged rats was previously observed [9]. Both selective COX-2 inhibition and nonselective COX inhibition (simultaneous COX-1 and COX-2 inhibition) attenuated the vasodilator net effect of prostanoids on acetylcholine-induced vascular relaxation, indicating the decreased participation of vasodilator prostanoids derived from COX-2 in vascular tone regulation. To some extent, this finding adds to the abovementioned results, which indicate a reduced share of some substances with a vasodilatory effect, which may be compensated by an increase in the production of NO from iNOS. These substances are vasodilators derived from COX-2. Surprisingly, iNOS did not modify vascular relaxation as it did in the contractile response to noradrenaline, indicating that the primary molecules responsible for vasodilation are prostanoids and not NO from iNOS.

Our research has shown that higher dietary copper intake (200%) did not have any impact on the levels of COX−1, COX−2, GAPDH, iCAM−1, HO−1, or eNOS in the blood serum of rats. COX-2 is widely recognized as the primary regulator of endothelial prostacyclin synthesis, which helps maintain the balance of thromboxane synthesis mediated by COX-1 in platelets. Therefore, specific inhibition of COX-2 is believed to lead to vasoconstriction and platelet aggregation, subsequently increasing the occurrence of cardiovascular events [21]. It has been widely observed that GAPDH is not just an average glycolytic enzyme. In fact, there is a growing body of evidence indicating that GAPDH has multiple functions. Its role as a mediator of cell death has also been emphasized. Over the past few years, numerous studies have indicated that a group of GAPDH molecules move to the nucleus in response to various stressors, many of which are linked to oxidative stress [22]. Unlike young rats, previous research on middle-aged rats showed that levels of COX-1, COX-2, and GAPDH were lower in the blood serum when the amount of dietary copper was doubled (200%) [9]. In our study, there was no change in TAS, which may be related to the lack of modification of iCAM1 concentration in the serum. ICAM-1 binds to lymphocyte function-associated antigen-1, an 82-integrin found on all leukocytes. Nonhematopoietic cells like fibroblasts, vascular ECs, and epithelial cells express ICAM-1 at low levels. Cytokines and endotoxins strongly upregulate ICAM-1 [23]. Endothelial dysfunction is characterized by increased expression of cellular adhesion molecules such as ICAM-1 and VCAM-1. These changes in the functional endothelium have been observed to be linked to barrier function, resulting in the movement of white blood cells and an increase in the contraction of blood vessels due to a decrease in the processing of substances that dilate blood vessels, like nitric oxide [24]. Various molecules within the ROS family play a role in regulating cellular functions, such as cell growth and intercellular adhesion molecules [24]. 

HO-1 is responsible for the oxidative breakdown of heme into biliverdin, carbon monoxide, and iron. Furthermore, the removal of toxic heme is just one of the many functions of HO-1, and its products have recently been acknowledged for their significant roles in various organs [25]. In our study, no change in HO-1 concentration in serum was observed, despite a significant change in copper concentration. Studies conducted on transgenic HO-1 mice and individuals with HO-1 deficiency have revealed the significant impact of HO-1 activity on a wide range of anatomical and functional processes. One of the key consequences of HO-1 deficiency is the damage to the vascular endothelium, which can result in the development of cardiovascular diseases [25]. In the livers of copper-deficient rats, there was an observed increase in HO-1 activity. Although the cause of the increase in HO-1 activity remains unclear, it is possible that alterations in iron and selenium metabolism during copper deficiency may play a role. Moreover, in copper-deficient rats, a decrease in the activity of copper-dependent antioxidant enzymes, such as superoxide dismutase (SOD)-1 and ceruloplasmin, leads to an increase in oxidative stress. This increase in oxidative stress may play a role in the activation of HO-1, an important enzyme in copper deficiency [26]. It is worth mentioning that HO-1 was the only enzyme mentioned above, which was higher in zinc-supplemented middle-aged rats [27]. In the same study, copper was lower, opposite to selenium and TAS, which were higher.

Reactive oxygen species (ROS) play a crucial role as reactive intermediates of molecular oxygen and serve as vital secondary messengers in cells. However, an imbalance between the production of ROS and the body’s antioxidant defense systems can lead to endothelial dysfunction. This dysfunction is a major contributor to vascular damage in metabolic and atherosclerotic diseases [28]. There are multiple biomarkers and methods of measurement available to assess antioxidant status. Recent studies have utilized the TAS, ACW, and ACL [8,29], and we also used this method. In a previous study, it was discovered that TAS was higher in middle-aged rats [9]. However, in the present study, neither TAS nor ACW, ACL in the blood, nor MDA, SOD, and CAT levels in the arteries and heart of the rats were altered. Surprisingly, the levels of NO, O_2_^•−^ and H_2_O_2_ were higher in the aortic rings. The existing scientific literature has not adequately explained the relationship between excess dietary copper and oxidative stress due to only a few studies have been conducted on this subject. Li et al. found that the ratio of reduced and oxidized glutathione (GSH/GSSG) of the copper treatment groups lowered as the copper concentration increased in porcine small intestinal epithelial cells. GSH, being an essential antioxidant, can undergo oxidation to GSSG during the oxidative stress induced by prolonged copper exposure, leading to a reduction in GSH levels [30]. These authors concluded that an increased concentration of copper results in oxidative stress. In addition, Galhardi et al. demonstrated that there are other consequences of copper exposure and diabetes, including elevated levels of ROS and changes in indicators of oxidative stress. Both diabetic rats and rats treated with 60 mg/kg copper showed elevated lipid hydroperoxide levels in the serum, but markedly reduced glutathione peroxidase and SOD activities [31]. Furthermore, Yildiz et al. demonstrated a significant reduction in superoxide dismutase (SOD) activity in the liver was observed in the group exposed to copper sulfate. Consequently, copper sulfate also induces oxidative alterations in the liver [32].

The liver and kidneys were chosen as these two vital organs were proven to be affected by microelement dysregulation [33]. In our study, copper levels were higher in the blood, liver, and kidneys of experimental rats, with no difference in zinc levels. Compensatory mechanisms strictly regulate the serum concentrations of copper and zinc, ensuring that they remain stable within specific ranges corresponding to nutritional intake [27]. Nevertheless, there are mechanisms in the blood that are designed to reduce the amount of zinc and increase the amount of copper during inflammatory conditions. As a result, a common characteristic of various chronic diseases associated with aging is an elevated copper-to-zinc ratio [34]. Moreover, in rat kidneys, potassium, and iron concentrations were higher, while calcium and chromium concentrations were lower. In the rat liver, magnesium levels were higher. Sodium, zinc, manganese, selenium, cobalt, molybdenum, and vanadium were not modified, which is difficult to discuss as there are no available data regarding mineral concentrations in the liver or kidneys of copper-supplemented mammals. It has been recently proven that the status of trace elements in metabolic disorders should be carefully analyzed for effective nutritional and therapeutic strategies [35]. In addition to metabolic disorders, some trace element imbalances may also dysregulate the other. For instance, chromium supplementation compensates for the copper content in iron deficiency [35]. Excessive iron intake induces systemic copper deficiency and hypercholesterolemia [36]. Exposure to copper, manganese, and mercury, when they exceed the safety limits, induces changes in neurological tissue [37], and under certain conditions, chromium can act as a pro-oxidant agent [38].

In our study, no significant differences were found in body weight gain, fat content, or lean body composition. In addition, there were no differences in the weights of the brain, heart, kidneys, liver, or spleen. Previous studies with middle-aged rats support the data presented in this article [9]. Consistent with our research, Filetti et al. (2023) reported that there were no significant differences in weight between the control group and the groups that were administered different amounts of copper [39]. However, animals exposed to copper sulfate had significantly lower body weights compared to those in the control group [40].

There are a few limitations in our study due to only a few prior research available and the reliance on self-reported data. While copper deficiency is a well-studied topic, there is a lack of research on its chronic exposure to low concentrations, even though environmental pollution, such as copper contamination in water from aged pipes, is becoming more prevalent. The addition of other mineral(s) to the daily diet, together with increased copper, should be considered in further studies. In addition, varying doses of copper should be studied in both young and aged rats to obtain more precise data for further discussion.

## 5. Conclusions

Our study revealed that supplementation with 200% of the recommended daily dietary quantity of copper did not modify vascular contraction in young rats. Vascular relaxation tended to increase, while the participation of vasodilator prostanoids was attenuated. Increased nitric oxide derived from iNOS may constitute a compensatory mechanism for the decreased effect of COX-2-derived vasodilator prostanoids. Among the minerals examined, copper, potassium, iron, calcium, chromium, and magnesium were modified. No significant differences were observed in the concentrations of sodium, zinc, manganese, selenium, cobalt, molybdenum, and vanadium between the studied groups in the rat plasma, rat kidneys, and liver. Detailed data on the status of trace elements and their interactions in patients of different age groups are strongly required for effective nutritional and therapeutic intervention.

## Figures and Tables

**Table 1:** The influence of COX and iNOS inhibition on the acetylcholine-induced vasodilation of isolated aortic rings from copper-supplemented young Wistar rats.

Group Copper Content		Control Conditions (CC) *			NS-398 **			NS-398 + SC-560 ***			1400 W ****		
		AUC	Emax (%)	pEC_50_	AUC	Emax (%)	pEC_50_	AUC	Emax (%)	pEC_50_	AUC	Emax (%)	pEC_50_
Group A 100% Cu	Mean	221.2	87.43	7.346	186.6 *	87.84	6.948	172.8 *	87.47	6.830	249.7	94.69	7.436
	SEM	22.26	3.695	0.131	23.76	4.658	0.139	18.36	3.883	0.112	16.09	2.391	0.082
Group B 200% Cu	Mean	252.4	94.31	7.576	248.7	92.71	7.498	206.1	85.60	7.306	226.0	91.35	7.346
	SEM	14.64	2.045	0.070	22.47	3.170	0.113	26.19	4.122	0.141	13.68	1.969	0.065
*p*		0.035	ns	ns	ns	ns	ns	ns	ns	ns	ns	ns	ns

Values are based on the concentration-response curves. Data are expressed as means ± SEM, *n* = 8, * *p* ≤ 0.05 compared with the control conditions as determined by two-way ANOVA followed by Tukey’s post hoc test. ** selective COX-2 inhibition with NS-398, 10 µM, *** selective COX-1 (SC-560, 10 µM) and COX-2 inhibition (NS-398, 10 µM), **** iNOS inhibition with 1400 W, 1 µM. Abbreviations: AUC—area under the curve; CC—control conditions; COX—cyclooxygenase; Cu—copper; iNOS—inducible nitric oxide synthase; ns—not significant.

## Data Availability

The original contributions presented in the study are included in the article/Supplementary Materials; further inquiries can be directed to the corresponding author.

## Supplementary Materials

The following supporting information can be downloaded at https://www.mdpi.com/article/10.3390/nu16193230/s1, Table S1: The ELISA kits used for the determination of antioxidant status in the presented study; Table S2: Results detected.

## References

[1] Chen L., Min J., Wang F. (2022). Copper homeostasis and cuproptosis in health and disease. Signal Transduct. Target. Ther..

[2] Wang T., Xiang P., Ha J.H., Wang X., Doguer C., Flores S.R.L., Kang Y.J., Collins J.F. (2018). Copper supplementation reverses dietary iron overload-induced pathologies in mice. J. Nutr. Biochem..

[3] Hajam Y.A., Rani R., Ganie S.Y., Sheikh T.A., Javaid D., Qadri S.S., Pramodh S., Alsulimani A., Alkhanani M.F., Harakeh S. (2022). Oxidative Stress in Human Pathology and Aging: Molecular Mechanisms and Perspectives. Cells.

[4] Kitala K., Tanski D., Godlewski J., Krajewska-Włodarczyk M., Gromadziński L., Majewski M. (2023). Copper and Zinc Particles as Regulators of Cardiovascular System Function—A Review. Nutrients.

[5] El-Ta’alu A., Ahmad M.M. (2022). Age-Dependent Effects of Copper Toxicity on Connective Tissue Structural Stability in Wistar Rats Skin. Niger. J. Physiol. Sci..

[6] Turnlund J.R., Reager R.D., Costa F. (1988). Iron and copper absorption in young and elderly men. Nutr. Res..

[7] Johnson P.E., Milne D.B., Lykken G.I. (1992). Effects of age and sex on copper absorption, biological half-life, and status in humans. Am. J. Clin. Nutr..

[8] Majewski M., Gromadziński L., Cholewińska E., Ognik K., Fotschki B., Juśkiewicz J. (2023). The Interaction of Dietary Pectin, Inulin, and Psyllium with Copper Nanoparticle Induced Changes to the Cardiovascular System. Nutrients.

[9] Kitala-Tańska K., Socha K., Juśkiewicz J., Krajewska-Włodarczyk M., Majewski M. (2024). The Effect of an Elevated Dietary Copper Level on the Vascular Contractility and Oxidative Stress in Middle-Aged Rats. Nutrients.

[10] Gordon C.J., Phillips P.M., Johnstone A.F. (2016). A noninvasive method to study regulation of extracellular fluid volume in rats using nuclear magnetic resonance. Am. J. Physiol. Renal. Physiol..

[11] Majewski M., Juśkiewicz J., Krajewska-Włodarczyk M., Gromadziński L., Socha K., Cholewińska E., Ognik K. (2021). The Role of 20-HETE, COX, Thromboxane Receptors, and Blood Plasma Antioxidant Status in Vascular Relaxation of Copper-Nanoparticle-Fed WKY Rats. Nutrients.

[12] Kitala-Tańska K. (2024). Summary of the Results. https://app.biorender.com/citation/66e2f1c5ea3e111ddbda34e8.

[13] Wang Y.C., Hu C.W., Liu M.Y., Jiang H.C., Huo R., Dong D.L. (2013). Copper induces vasorelaxation and antagonizes noradrenaline-induced vasoconstriction in rat mesenteric artery. Cell. Physiol. Biochem..

[14] Kunutsor S.K., Dey R.S., Laukkanen J.A. (2021). Circulating Serum Copper Is Associated with Atherosclerotic Cardiovascular Disease, but Not Venous Thromboembolism: A Prospective Cohort Study. Pulse.

[15] Li L., Shi J., Liu W., Luo Y., Gao S., Liu J.X. (2024). Copper overload induces apoptosis and impaired proliferation of T cell in zebrafish. Aquat. Toxicol..

[16] Tanaka A., Kaneto H., Miyatsuka T., Yamamoto K., Yoshiuchi K., Yamasaki Y., Shimomura I., Matsuoka T.A., Matsuhisa M. (2009). Role of copper ion in the pathogenesis of type 2 diabetes. Endocr. J..

[17] Ferns G.A., Lamb D.J., Taylor A. (1997). The possible role of copper ions in atherogenesis: The Blue Janus. Atherosclerosis.

[18] Chen Z., Li Y.Y., Liu X. (2023). Copper homeostasis and copper-induced cell death: Novel targeting for intervention in the pathogenesis of vascular aging. Biomed. Pharmacother..

[19] Yan M., Liu D.L., Chua Y.L., Chen C., Lim Y.L. (2001). Effects of micromolar concentrations of manganese, copper, and zinc on alpha1-adrenoceptor-mediating contraction in rat aorta. Biol. Trace Elem. Res..

[20] Cuzzocrea S., Persichini T., Dugo L., Colasanti M., Musci G. (2003). Copper induces type II nitric oxide synthase in vivo. Free. Radic. Biol. Med..

[21] Luo W., Liu B., Zhou Y. (2016). The endothelial cyclooxygenase pathway: Insights from mouse arteries. Eur. J. Pharmacol..

[22] Hara M.R., Cascio M.B., Sawa A. (2006). GAPDH as a sensor of NO stress. Biochim. Biophys. Acta.

[23] Couffinhal T., Duplàa C., Moreau C., Lamazière J.M., Bonnet J. (1994). Regulation of vascular cell adhesion molecule-1 and intercellular adhesion molecule-1 in human vascular smooth muscle cells. Circ. Res..

[24] Habas K., Shang L. (2018). Alterations in intercellular adhesion molecule 1 (ICAM-1) and vascular cell adhesion molecule 1 (VCAM-1) in human endothelial cells. Tissue Cell..

[25] Loboda A., Jazwa A., Grochot-Przeczek A., Rutkowski A.J., Cisowski J., Agarwal A., Jozkowicz A., Dulak J. (2008). Heme oxygenase-1 and the vascular bed: From molecular mechanisms to therapeutic opportunities. Antioxid. Redox Signal..

[26] Johnson W.T., DeMars L.C. (2004). Increased heme oxygenase-1 expression during copper deficiency in rats results from increased mitochondrial generation of hydrogen peroxide. J. Nutr..

[27] Borkowska-Sztachańska M., Thoene M., Socha K., Juśkiewicz J., Majewski M.S. (2024). Decreased vascular contraction and changes in oxidative state in middle-aged Wistar rats after exposure to increased levels of dietary zinc. Toxicol. Appl. Pharmacol..

[28] Incalza M.A., D’Oria R., Natalicchio A., Perrini S., Laviola L., Giorgino F. (2018). Oxidative stress and reactive oxygen species in endothelial dysfunction associated with cardiovascular and metabolic diseases. Vascul. Pharmacol..

[29] Karaaslan F., Demir F., Yılmaz R., Akıl E. (2023). Total oxidant/antioxidant status, copper and zinc levels in acute ischemic stroke patients after mechanical thrombectomy. Clin. Neurol. Neurosurg..

[30] Li R., Wen Y., Lin G., Meng C., He P., Wang F. (2020). Different Sources of Copper Effect on Intestinal Epithelial Cell: Toxicity, Oxidative Stress, and Metabolism. Metabolites.

[31] Galhardi C.M., Diniz Y.S., Faine L.A., Rodrigues H.G., Burneiko R.C.M., Ribas B.O., Novelli E.L. (2004). Toxicity of copper intake: Lipid profile, oxidative stress and susceptibility to renal dysfunction. Food Chem. Toxicol..

[32] Yildiz M., Boyacioglu M., Avcioglu M., Elmas S. (2023). Changes Induced by Copper Toxicity in the Rat Liver and the Effects of Panax Ginseng on These Changes. Biol. Bull. Russ. Acad Sci..

[33] Zou Y., Wu S., Xu X., Tan X., Yang S., Chen T., Zhang J., Li S., Li W., Wang F. (2024). Cope with copper: From molecular mechanisms of cuproptosis to copper-related kidney diseases. Int. Immunopharmacol..

[34] Malavolta M., Piacenza F., Basso A., Giacconi R., Costarelli L., Mocchegiani E. (2015). Serum copper to zinc ratio: Relationship with aging and health status. Mech. Aging Dev..

[35] Staniek H. (2019). The Combined Effects of Cr(III) Supplementation and Iron Deficiency on the Copper and Zinc Status in Wistar Rats. Biol. Trace Elem. Res..

[36] Lee J., Jang H., Doo M., Kim B.H., Ha J.H. (2024). High Iron Consumption Modifies the Hepatic Transcriptome Related to Cholesterol Metabolism. J. Med. Food..

[37] Draper M., Bester M.J., Van Rooy M.J., Oberholzer H.M. (2023). Adverse neurological effects after exposure to copper, manganese, and mercury mixtures in a Spraque-Dawley rat model: An ultrastructural investigation. Ultrastruct. Pathol..

[38] Majewski M., Gromadziński L., Cholewińska E., Ognik K., Fotschki B., Juśkiewicz J. (2022). Dietary Effects of Chromium Picolinate and Chromium Nanoparticles in Wistar Rats Fed with a High-Fat, Low-Fiber Diet: The Role of Fat Normalization. Nutrients.

[39] Filetti F.M., Schereider I.R.G., Wiggers G.A., Miguel M., Vassallo D.V., Simões M.R. (2023). Cardiovascular Harmful Effects of Recommended Daily Doses (13 µg/kg/day), Tolerable Upper Intake Doses (0.14 mg/kg/day) and Twice the Tolerable Doses (0.28 mg/kg/day) of Copper. Cardiovasc. Toxicol..

[40] Kumar V., Kalita J., Misra U.K., Bora H.K. (2015). A study of dose response and organ susceptibility of copper toxicity in a rat model. J. Trace Elem. Med. Biol..

