# Supplementary material for: Prolonged Copper Supplementation Modified Minerals in the Kidney, Liver and Blood, and Potentiated Oxidative Stress and Vasodilation of Isolated Aortic Rings in Young Wistar Rats
Source: Nutrients. 2024 Sep 24;16(19):3230. doi: 10.3390/nu16193230 (PMC11478114; doi:10.3390/nu16193230)
Supplement: Supplementary file 1 [file nutrients-16-03230-s001.zip › Table S2.pdf]

Table S2. Results detected.

	100% Cu (Group A)		200% Cu (Group B)				
	Mean	"Std. Deviation"	Mean	"Std. Deviation"	x–fold (B/A)	Δ	p value
COX–1$_{(b)}$ (ng/mL)	1.823	0.607	1.315	0.285	0.72	-0.28	0.291
COX–2$_{(b)}$ (pg/mL)	101.6	18.50	82.0	19.22	0.81	-0.19	0.324
GAPDH$_{(b)}$ (ng/mL)	0.123	0.090	0.097	0.041	0.78	-0.22	0.334
HO–1$_{(b)}$ (ng/mL)	1.409	0.364	1.663	0.351	1.18	0.18	0.156
NOS3$_{(b)}$ (pg/mL)	92.30	21.97	93.88	23.23	1.02	0.02	>0.999
iCAM$_{(b)}$ (pg/mL)	72.01	18.12	97.03	19.12	1.35	0.35	0.435
MDA$_{(h)}$ (μmol/kg)	3.408	1.151	3.291	1.249	0.97	-0.03	>0.999
MDA$_{(b)}$ (μmol/L)	1.721	0.328	1.823	0.875	1.06	0.06	0.535
CAT$_{(h)}$ (U/g protein)	449.9	92.79	456.8	149.4	1.02	0.02	>0.999
CAT$_{(b)}$ (U/mL)	105.1	34.02	88.11	23.11	0.84	-0.16	0.364
SOD$_{(h)}$ (U/g protein)	605.4	115.5	590.0	63.98	0.97	-0.03	0.998
SOD$_{(b)}$ (U/mL)	48.31	9.02	55.12	11.00	1.14	0.14	0.342
ACW$_{(b)}$ (μg/mL ascorbic acid)	2.946	0.717	2.859	0.840	0.97	-0.03	0.989
ACL$_{(b)}$ (μg/mL Trolox)	3.637	0.326	3.571	0.265	0.98	-0.02	0.969
TAS$_{(b)}$ (mmol/L)	0.500	0.461	0.559	0.116	1.12	0.12	0.179
**Cu$_{(l)}$ (μg/g)**	**3.110**	**0.180**	**3.579**	**0.522**	**1.15**	**0.15**	**0.032**
**Cu$_{(k)}$ (μg/g)**	**6.255**	**0.907**	**7.707**	**1.676**	**1.23**	**0.23**	**0.045**
K$_{(l)}$ (μg/g)	2859	191.2	2888	223.6	1.01	0.01	0.753
**K$_{(k)}$ (μg/g)**	**2700**	**105.2**	**2909**	**145.9**	**1.08**	**0.08**	**0.001**
Fe$_{(l)}$ (μg/g)	109.1	14.36	115.9	23.08	1.06	0.06	0.609
**Fe$_{(k)}$ (μg/g)**	**93.02**	**9.633**	**104.8**	**26.40**	**1.13**	**0.13**	**0.046**
Ca$_{(l)}$ (μg/g)	35.89	5.896	37.95	6.839	1.06	0.06	0.293
**Ca$_{(k)}$ (μg/g)**	**82.00**	**11.23**	**71.92**	**8.514**	**0.88**	**-0.12**	**0.001**
Cr$_{(l)}$ (ng/g)	24.05	8.916	19.8	8.730	0.82	-0.18	0.637
**Cr$_{(k)}$ (ng/g)**	**62.91**	**18.14**	**48.29**	**14.23**	**0.77**	**-0.23**	**0.005**
**Mg$_{(l)}$ (μg/g)**	**189.0**	**14.33**	**199.4**	**20.38**	**1.06**	**0.06**	**0.012**
Mg$_{(k)}$ (μg/g)	175.9	15.42	178.1	14.96	1.01	0.01	0.925
Na$_{(l)}$ (μg/g)	540.4	44.06	567.5	95.31	1.05	0.05	0.193
Na$_{(k)}$ (μg/g)	1034	205.9	962.3	136.6	0.93	-0.07	0.182
Zn$_{(l)}$ (μg/g)	23.35	1.273	24.55	2.134	1.05	0.05	0.085
Zn$_{(k)}$ (μg/g)	23.02	1.388	24.43	1.708	1.06	0.06	0.135
Mn$_{(l)}$ (μg/g)	1.944	0.221	1.909	0.264	0.98	-0.02	0.999
Mn$_{(k)}$ (μg/g)	0.9046	0.101	0.9170	0.135	1.01	0.01	0.359
Se$_{(l)}$ (ng/g)	756.1	62.28	762.4	83.30	1.01	0.01	0.894
Se$_{(k)}$ (ng/g)	1886	122.6	1830	234.3	0.97	-0.03	0.806
Co$_{(l)}$ (ng/g)	6.526	2.126	5.008	2.592	0.77	-0.23	0.107
Co$_{(k)}$ (ng/g)	64.56	10.95	59.16	11.82	0.92	-0.08	0.140
Mo$_{(l)}$ (ng/g)	357.1	46.52	373.4	67.32	1.05	0.05	0.210
Mo$_{(k)}$ (ng/g)	237.6	18.42	247.8	24.96	1.04	0.04	0.156
V$_{(l)}$ (ng/g)	5.114	0.834	4.428	0.801	0.87	-0.13	0.119
V$_{(k)}$ (ng/g)	14.38	2.717	13.95	2.394	0.97	-0.03	0.965
**b – blood plasma**<br>**h – heart**<br>**l – liver**<br>**k – kidney**							
